# Structural, Evolutionary, and Regulatory Divergence of *FAH12* from *FAD2* Reveals Recurrent Independent Neofunctionalization Underlying Ricinoleic Acid Biosynthesis in *Ricinus communis*

**DOI:** 10.3390/plants15101544

**Published:** 2026-05-19

**Authors:** Fanqing Meng, Jing Sun, Zekun Zhou, Guofang Yuan, Bin Tian, Aizhong Liu, Anmin Yu

**Affiliations:** 1 Key Laboratory for Forest Resources Conservation and Utilization in the Southwest Mountains of China, Ministry of Education, Southwest Forestry University, Kunming 650224, China; mengfanqing@swfu.edu.cn (F.M.); zhouzekun@swfu.edu.cn (Z.Z.);; 2 Yunnan Key Laboratory of Plateau Wetland Conservation, Restoration and Ecological Services, Southwest Forestry University, Kunming 650224, China; tianbin@swfu.edu.cn; 3 National Plateau Wetlands Research Center, Southwest Forestry University, Kunming 650224, China; 4 Yunnan Provincial Key Laboratory for Conservation and Utilization of In-forest Resource, Southwest Forestry University, Kunming 650224, China; 5 Yunnan Key Laboratory of Crop Wild Relatives Omics, Kunming Institute of Botany, Chinese Academy of Sciences, Kunming 650201, China

**Keywords:** *Ricinus communis*, FAH12, FAD2, hydroxy fatty acid, gene duplication, recurrent independent neofunctionalization, transcriptional regulation

## Abstract

*Ricinus communis* is the primary commercial source of ricinoleic acid (RA), a hydroxy fatty acid (HFA) synthesized by the fatty acid hydroxylase *FAH12*, which evolved from the Δ12-oleate desaturase *FAD2*. However, the evolutionary origins and diversification mechanisms of *FAH12* across HFA-producing plants remain poorly understood. Here, we performed a comprehensive cross-species analysis of *FAH12* and *FAD2* homologs by integrating sequence analysis, structural modeling, phylogenetic reconstruction, and transcriptomic profiling. Across all currently available HFA-producing plant lineages, we found that amino acid substitutions associated with hydroxylase activity exhibit strong lineage-specific patterns rather than universal conservation, indicating multiple evolutionary solutions to catalytic divergence. Phylogenetic and synteny analyses further suggest that *FAH12* arose independently from ancestral *FAD2* duplications in distinct plant lineages, supporting a model of recurrent independent neofunctionalization. Transcriptomic and qRT-PCR analyses reveal that *FAH12* exhibits a highly specialized endosperm-preferential expression pattern and is embedded within a regulatory network that is partially decoupled from that of *FAD2*. Together, these findings demonstrate that *FAH12* evolution is driven by recurrent independent origins coupled with transcriptional specialization, providing a framework linking structural variation, evolutionary history, and regulatory divergence for understanding and engineering hydroxy fatty acid biosynthesis in plants.

## 1. Introduction

Fatty acids constitute fundamental building blocks of plant membrane lipids and storage reserves, playing vital roles in maintaining membrane fluidity, energy storage, and mediating cellular signaling processes [1]. In higher plants, desaturation and structural modification of fatty acids generate a wide diversity of triacylglycerol (TAG) compositions, thereby supporting various physiological processes and expanding the industrial applications of vegetable oils [2]. Many seed oils contain unusual fatty acids featuring modifications such as hydroxylation, epoxidation, chain-length variation (≥22 or ≤12 carbons), unsaturation changes, or stereochemical shifts [3], resulting in distinct physicochemical properties and specialized industrial uses. In particular, hydroxy fatty acids (HFAs)-rich oils have been widely applied in bio-lubricants, biodegradable polymers, sustainable coatings, biomedical materials, and biodiesel production due to their unique functional properties [4]. Notably, processing techniques such as degumming have been shown to alter the structural profiles and physicochemical properties of phospholipids from oil-producing plants like Idesia polycarpa, further highlighting the industrial relevance of HFA-related research [5]. However, HFAs accumulate in only a limited number of species, including castor (*Ricinus communis* L.), Lesquerella (*Physaria fendleri*), *Orychophragmus violaceus*, *Hiptage benghalensis*, and the fungi *Claviceps purpurea* [6,7,8,9,10,11]. Recent genomic and functional studies have begun to elucidate the molecular basis of HFA accumulation in these species, including the identification of key amino acid residues governing *FAH12* activity in Physaria and the development of biotransformation strategies to produce novel di- and tri-hydroxy fatty acids from plant oils [12,13].

These hydroxylation reactions, producing HFA (12-hydroxy-9-octadecenoic acid), occur at the Δ12 position of oleic acid (C18:1-OH). It is generally accepted that HFAs are synthesized from oleic acid (C18:1) via a specific fatty acid hydroxylase that introduces a hydroxyl group at the Δ12 carbon. In particular, castor oil, extracted from castor bean seeds, is predominantly composed of HFAs and exhibits unique chemical properties that are extensively exploited in industrial applications [14]. The gene encoding the Δ12 fatty acid hydroxylase, *RcFAH12*, was identified in castor bean, as a homolog of *RcFAD2* (*fatty acid desaturase 2*), which catalyzes desaturation at the same carbon position [9]. Typically, *FAD2* enzymes convert oleic acid (C18:1) to linoleic acid (C18:2) in major oilseed crops such as rapeseed (*Brassica napus* L.), sunflower (*Helianthus annuus* L.), and peanut (*Arachis hypogaea*) [15,16,17]. Despite their distinct catalytic functions as hydroxylase (*FAH12*) and desaturase (*FAD2*), these enzymes exhibit high sequence similarity in castor bean [18]. Both FAH12 and FAD2 are endoplasmic reticulum (ER)-localized membrane-associated that utilize oleic acid (C18:1) as a substrate and function through a conserved di-iron catalytic mechanism, requiring molecular oxygen, cytochrome b5 (cyt b5), and cyt b5 reductase [19]. FAH12 and FAD2 share conserved desaturase domains (PF00487 and PF11960) and three characteristic His-box motifs [4]. Notably, previous studies identified seven key amino acid residues that may influence enzyme activity and determine whether the enzyme catalyzes desaturation (FAD2) or hydroxylation (FAH12) at the Δ12 position [20]. However, it remains unclear whether these critical amino acid residues are conserved across *FAD2s* and *FAH12s* homologs in different species. Although *FAH12s* are predominantly expressed in seeds and are closely associated with HFA biosynthesis in developing oilseeds of castor, lesquerella and *H. benghalensis* [6,8,9], their transcriptional regulation remains poorly understood. Several transcription factors, including WRI1 (WRINKLED1), LEC1 (LEAFY COTYLEDON1), LEC2, and ABI5 (ABSCISIC ACID INSENSITIVE5), have been identified as key regulators of fatty acid biosynthesis and oil accumulation in developing seeds [21,22,23,24]. However, whether these regulators directly control FAH12 expression has not been clearly established. In addition, phylogenetic studies suggest that *FAH12* genes originated from *FAD2* via gene duplication events in both castor and Lesquerella [6,9,25]. Nevertheless, the evolutionary trajectory and divergence mechanisms of *FAH12* and *FAD2* remain largely unresolved.

In this study, we performed comprehensive analyses of sequence variation, structural features, and phylogeny relationships between *FAH12* genes and their homologous *FAD2* genes across HFA-producing oilseed species. Based on the comparative transcriptomic data, we provide new insights into the structural, functional, and evolutionary divergence of *FAH12* and *FAD2* in plants. Furthermore, by identifying lineage-specific catalytic residues, expression characteristics, and candidate regulatory factors associated with hydroxylase activity, this study provides molecular insights that may facilitate future metabolic engineering strategies for hydroxy fatty acid production in conventional oilseed crops.

## 2. Results

### 2.1. Identification of FAD2s and FAH12s in Oilseed Crops Producing HFA-Oils

Since HFA-oils accumulate in only a limited number of plant species, including castor, *O. violaceus*, *P. fendleri*, *P. lindheimeri*, *P. auriculata*, and *H. benghalensis*, we systematically surveyed FAH12s and their homologous FAD2s in these species based on available genomic resources or previously characterized sequences [8,9,22,23,24,25,26,27]. To further investigate sequence similarity and phylogenetic relationships, additional representative species closely related to HFA-producing taxa were included. These comprised species closely related to castor, including *Discocleidion rufescens*, *Speranskia tuberculata*, as well as other Euphorbiaceae members such as *Jatropha curcas* L., *Manihot esculenta* Crantz, *Hevea brasiliensis*, and *Vernicia fordii*. In addition, species phylogenetically related to *O. violaceus* and *P. fendleri*, including *Arabidopsis thaliana* and *Brassica napus* L. from Brassicaceae, were incorporated, with maize (*Zea mays*) serving as a monocot reference. In total, 34 *FAD2* and 7 *FAH12* genes were identified across these species. All candidate sequences were confirmed by the presence of conserved domains PF00487 and PF11960 (Tables S1 and S2). The coding sequence (CDS) lengths ranged from 711 bp (*OvFAD2.4*) to 1296 bp (*HbFAD2.1*), encoding proteins of 236–431 amino acids with predicted molecular weights of 27.06–49.54 kDa (Table S3) and the pI values ranging from 6.64 (*HibFAD2.4*) to 9.00 (*PlFAH12*) (Table S3). The FA desaturase domain spanned 129–262 aa in length, while the DUF3474 domain ranged from 36 to 144 aa in length (Table S3). Most *FAD2* and *FAH12* proteins contained both the canonical FA_desaturase and DUF3474 domains, whereas only four *FAD2* genes (*BnFAD2.1*, *BnFAD2.3*, *OvFAD2.1* and *OvFAD2.4*) possessed only the FA_desaturase domain (Table S3 and Figures S1–S3). Prediction of subcellular localization indicated that all *FAD2* and *FAH12* were localized to the endoplasmic reticulum (ER) (Table S3), consistent with their established roles in phospholipid desaturation. Analysis of gene structure revealed that 19 genes were intronless, 14 genes contained a single intron, *OvFAH12* contained three introns, and *ZmFAD2.2* contained six introns. Despite minor variation in exon–intron organization, the overall conservation of domain organization and subcellular localization suggests that *FAD2s* and *FAH12s* share a common structural framework. Taken together, these results establish a conserved molecular basis across species, providing a foundation for subsequent analyses of functional and evolutionary divergence.

### 2.2. Identification of Key Amino Acid Residues Divergence Between FAD2s and FAH12s

**Figure 1:**
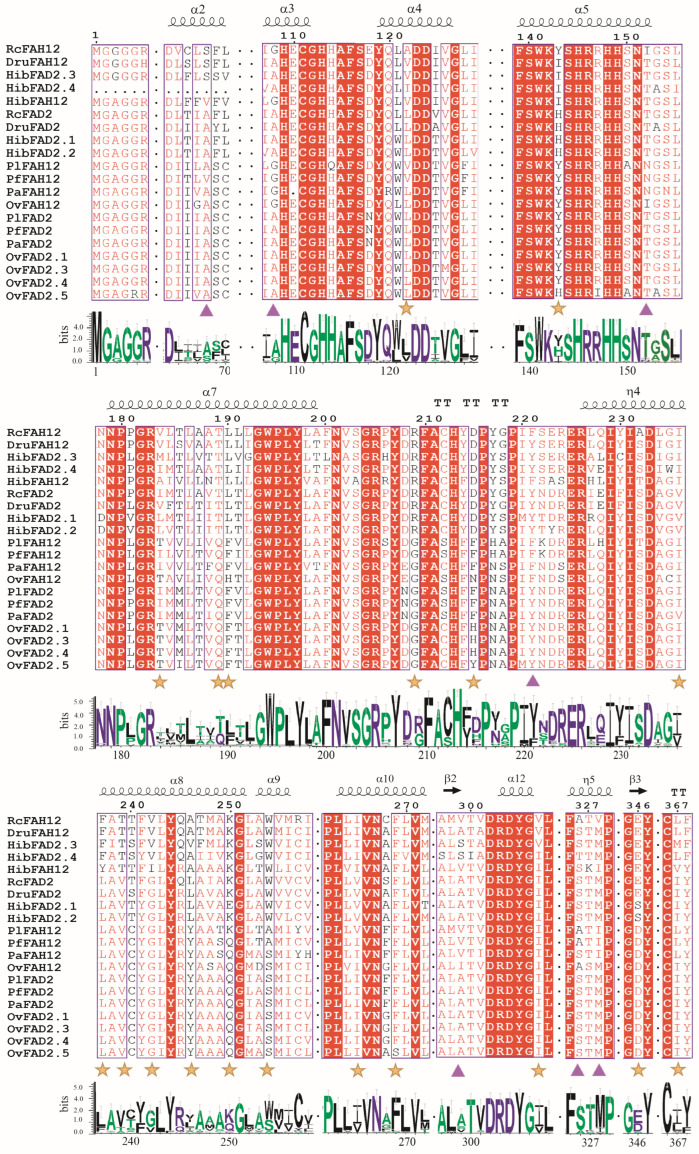
Multiple sequence alignment of canonical fatty acid desaturase (*FAD2*) and fatty acid hydroxylase 12 (*FAH12*). The alignment highlights conserved structural features and key amino acid differences underlying the functional divergence between *FAD2* and *FAH12*. Secondary structural elements (α-helices and β-strands) are annotated above the alignment. Seven previously reported diagnostic residues distinguishing *FAD2* from *FAH12* are marked with purple triangles. Nineteen newly identified diagnostic sites associated with hydroxylase specialization across Euphorbiaceae are labeled with yellow stars. Sequence logo plots below the alignment visualize conservation patterns and aa substitutions at these diagnostic positions, illustrating their contribution to the biochemical differentiation between desaturation and hydroxylation activities.

To evaluate whether the seven previously reported amino acid residues associated with catalytic divergence between *FAD2* and *FAH12* are conserved across species [20], we performed multiple sequence alignment of seven *FAH12* proteins (including *RcFAH12*, *DruFAH12*, *HibFAH12*, *PlFAH12*, *DfFAH12*, *PaFAH12*, and *OvFAH12*) together with fourteen homologous *FAD2* proteins from representative taxa (Figure 1). In addition, a comprehensive multiple sequence alignment of all FAD2 and FAH12 proteins identified in this study is provided in Figure S4. The alignment, combined with secondary structure prediction, revealed that all proteins shared a highly conserved structural framework composed of 15 α-helices, five β-strands, and four transmembrane domains (TM1–TM4). Three conserved histidine-rich motifs (H-box I–III: HECGH, HRRHH, and HVAHH) were identified in all sequences, consistent with their essential role in coordinating the di-iron catalytic center (Figure 1). Despite the strong conservation of the catalytic core, sequence variations were observed at specific amino acid positions outside the histidine motifs, suggesting that these residues may modulate enzyme specificity rather than directly affecting catalytic activity. Structural modeling further indicated that *RcFAD2* and *RcFAH12* adopted a conserved α-helical architecture, with the three histidine motifs consistently located within helices α3, α5, and α13 (Figure 1). Collectively, these findings indicate that the catalytic machinery is structurally stable, whereas peripheral residues likely contribute to functional diversification through modulation of substrate recognition and reaction specificity.

Comparative sequence analysis across Brassicales (e.g., *A. thaliana*, *B. napus*, *O. violaceus*, *P. fendleri*, *P. lindheimeri*, and *P. auriculata*) and Malpighiales (including *R. communis*, *D. rufescens*, and *H. benghalensis*) revealed that the seven previously proposed “diagnostic residues” (corresponding to substitutions A67S, A108G, T152N/I, Y221F, A299V/S, S326A/S, and M328I in *RcFAH12* relative to *RcFAD2*) exhibited lineage-specific substitution patterns rather than universal conservation [18,20]. Our expanded dataset further demonstrates that, although substitutions at these positions distinguish *FAD2* from *FAH12*, their patterns are not conserved across all lineages but instead follow clear order-specific evolutionary trajectories. For example, in Brassicales, both protein types predominantly retain A at position 67, with S observed only in *BnFAD2.2*. In contrast, Malpighiales frequently display an A→S shift between *FAD2* and *FAH12*, with *HibFAH12* uniquely harboring V at this position, underscoring its diagnostic value within this order (Figure 1). At position 108, Brassicales homologs typically exhibit an A→G substitution between *FAD2* and *FAH12*, whereas Malpighiales proteins largely retain A, with G restricted to *RcFAH12* and *HibFAH12*. Similarly, at position 152, Brassicales *FAH12* homologs commonly show a T→N substitution relative to *FAD2*, while Malpighiales proteins generally retain T, except for *RcFAH12*, which carries I. At position 221, *FAH12* proteins in Brassicales consistently contain F instead of Y (present in *FAD2*), whereas both enzyme types in Malpighiales predominantly retain Y, with F occurring only in *RcFAH12* and *HibFAH12* (Figure 1). Collectively, positions 67, 108, and 152 contribute substantially to *FAH12*–*FAD2* divergence in Brassicales and castor. At position 299, substitutions clearly distinguish *FAH12* from *FAD2* across both orders: *FAH12* sequences predominantly carry S (Malpighiales) or V (Brassicales), whereas all *FAD2* proteins uniformly retain A. At position 326, all *FAD2* sequences contain S, whereas *FAH12* proteins exhibit lineage-specific variation (A in Brassicales and predominantly S in Malpighiales). At position 328, all *FAD2* proteins retain M, whereas *FAH12* proteins show order-specific substitutions (I in Brassicales and M or variants in Malpighiales). Together, these findings indicate that amino acid substitutions underlying *FAD2*–*FAH12* divergence are strongly lineage-dependent and reflect distinct evolutionary trajectories across angiosperm orders.

Beyond the seven canonical sites, we identified 19 additional residues exhibiting diagnostic substitution patterns associated with functional specialization between *FAD2* and *FAD2*-*like* proteins (including *FAH12*) (Figure 1 and Figure S4). Among these, nine positions (122, 143, 236, 237, 239, 242, 246, 265, and 307) displayed distinct residue shifts (e.g., L→V, H→I, V→I, L→F, A→T, G→V, L→A, V→I, and I→V) that differentiate *FAD2* and *FAH12* within Malpighiales. In Brassicales, position 185 (M→V) serves as a robust diagnostic marker distinguishing the two protein types (Figure 1). Notably, position 367 shows a consistent I→L substitution across both orders, suggesting a shared evolutionary trajectory and possible structural adaptation. Additionally, five positions (189–190, 209, 250, 254, and 346) exhibit consistent differences between Brassicales and Malpighiales (QF→TL, G→R, Q→K, S→W, and D→E) (Figure 1), indicating their potential as lineage-specific markers. Furthermore, position 269 (W→F) differentiates monocots from dicots, while position 215 distinguishes subgroups within Brassicales, with *A. thaliana* and *P. fendleri* carrying F, and *O. violaceus* and *B. napus* harboring H. Overall, our results demonstrate that the seven historically defined diagnostic residues do not constitute a universally conserved mechanism governing hydroxylase activity [20]. Instead, *FAD2*–*FAH12* divergence reflects a more complex evolutionary landscape, in which multiple lineage-dependent amino acid substitutions collectively contribute to functional specialization. These 19 newly identified residues substantially expand the repertoire of candidate determinants underlying the transition from desaturation to hydroxylation. While these 19 residues represent strong candidates for functional assays, experimental validation through site-directed mutagenesis and heterologous expression will be required to definitively establish their individual and combinatorial contributions to the transition from desaturation to hydroxylation. Moreover, these lineage-specific signatures provide valuable molecular markers for reconstructing the evolutionary diversification of fatty acid–modifying enzymes across angiosperms.

### 2.3. Structural Insights into Catalytic Divergence Through 3D Modeling and Active-Site Pocket Analysis

**Figure 2:**
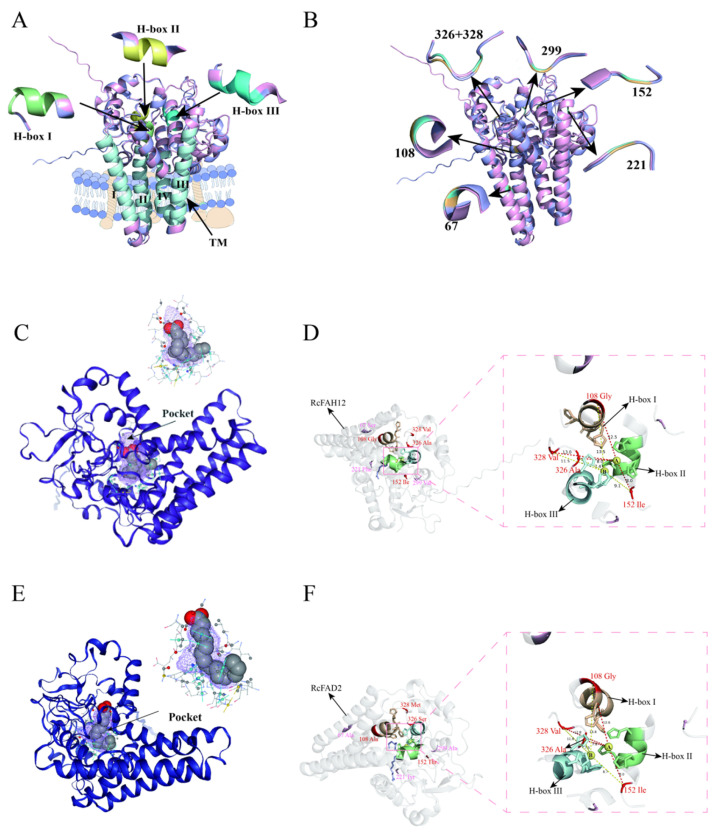
Structural basis underlying the catalytic divergence between *RcFAD2* and *RcFAH12*. (**A**) Superimposed three-dimensional models of *RcFAD2* (green ribbons) and *RcFAH12* (purple ribbons) reveal a highly conserved membrane-anchored architecture comprising four transmembrane helices (TM I–IV, labeled). The three histidine boxes (H-box I–III, sticks) converge around the di-iron catalytic core at the active site in both enzymes. The lipid bilayer is schematically represented as blue circles. (**B**) Surface view representing the seven diagnostic amino acid residues (spheres labeled by their positions: 67, 108, 152, 221, 299, 326, 328) that differ between *RcFAD2* and *RcFAH12*. These residues line the substrate entry channel and likely reshape the catalytic microenvironment, influencing substrate positioning and reaction outcome while preserving the overall protein fold. (**C**) Molecular docking of oleic acid (C18:1, gray/red spheres) into *RcFAH12* illustrates the substrate orientation and interactions with surrounding catalytic residues (displayed as stick models, with heteroatoms colored by element). The substrate is enclosed within the binding pocket, outlined by the purple mesh contour, favoring hydroxylation at the C12 position. (**D**) Visualization of the *RcFAH12* active-site cavity (gray ribbon) depicts a larger and more flexible binding pocket (249.34 Å^3^), providing structural space that accommodates a substrate conformation compatible with hydroxylation chemistry. (**E**) Docking results for *RcFAD2* (blue ribbon) show a distinct substrate-binding mode, where oleic acid (C18:1, purple spheres/sticks) interacts with nearby residues (purple sticks) consistent with canonical desaturation. (**F**) Surface representation of *RcFAD2* demonstrates a more compact and hydrophobic catalytic pocket (201.22 Å^3^), restricting substrate orientation to promote hydrogen abstraction and double-bond formation during the desaturation reaction.

Predicted tertiary structures of *RcFAD2* and *RcFAH12* revealed a canonical triangular configuration formed by three conserved histidine motifs encircling the di-iron catalytic center, positioned above a membrane-anchored hydrophobic scaffold (Figure 1). Although the two enzymes shared 73.89% overall sequence identity (Figure S5), several amino acid substitutions located outside the histidine boxes and within transmembrane regions may potentially contribute to their divergent catalytic properties. *RcFAH12* was relatively conserved within Euphorbiaceae, exhibiting 82.60% identity with *DruFAH12* but only 63.61% with *PlFAH12* (Figure S6), suggesting lineage-specific divergence among hydroxylases. AlphaFold3 models of *RcFAD2* and *RcFAH12* displayed high confidence, with >90% of residues showing predicted local distance difference test (pLDDT) scores above 90 and strong template-modeling (pTM) values, supporting the reliability of these structural models for comparative interpretation (Figure S7). Structural superimposition revealed a nearly identical backbone fold for *RcFAD2* and *RcFAH12*, indicating that the seven distinguishing residues did not disrupt the global protein architecture (Figure 2A,B). However, these substitutions are likely to influence catalytic behavior by modulating the geometry and physicochemical properties of the substrate-binding pocket rather than altering the overall fold.

In silico pocket analysis and substrate docking predicted that *RcFAH12* possesses a larger and structurally adaptable substrate tunnel (249.34 Å^3^) compared with *RcFAD2* (201.22 Å^3^), which may reflect an expanded catalytic cavity potentially compatible with hydroxylation reactions (Figure 2C,E, Table S4). Similarly, *FAH12* proteins showed computationally predicted larger binding pocket volume than their corresponding *FAD2* homologs in *O. violaceus*, *D. rufescens*, and *H. benghalensis* (Figure S8). Collectively, these computational observations suggest that the seven key residues may collectively contribute to physicochemical changes within the catalytic pocket rather than inducing major structural rearrangements. For example, the T→I (position 152) and A→V (299) substitutions introduce bulkier hydrophobic side chains, potentially increasing local hydrophobicity and slightly expanding pocket volume (Figure 2B). The Y→F (221) and S→A (326) substitutions remove hydroxyl groups, which may weaken local hydrogen-bond interactions and relax steric constraints, thereby creating a more permissive substrate environment. The A→G substitution at position 108 may enhance flexibility in a loop region adjacent to the catalytic pocket, potentially facilitating conformational adjustments required for substrate positioning (Figure 2B). Additional variations, such as V→A (67) and M→V (328), may further fine-tune hydrophobic packing and could influence pocket dynamics as suggested by structural modeling.

Distance mapping showed that most diagnostic residues are located 7–13 Å from the di-iron center (FeA and FeB), placing them in positions that could potentially influence substrate orientation and the local redox microenvironment without directly coordinating the metal ions (Figure 2D,F). Although the absolute distances to the catalytic centers are similar between *RcFAD2* and *RcFAH12*, the combined effects—reduced polarity, increased hydrophobic volume, and altered side-chain flexibility—may reshape the substrate-binding cavity and could modulate substrate positioning relative to the catalytic center. Such changes are consistent with a modeled trend favoring hydroxylation in *RcFAH12* rather than the canonical Δ12-desaturation catalyzed by *RcFAD2* (Figure 2D,F). Taken together, these computational modeling results suggest that functional divergence between *FAD2* and *FAH12* is unlikely to arise from large-scale structural rearrangement, but instead appears to be associated with coordinated microenvironmental changes within the catalytic pocket. The conserved protein scaffold is maintained, while evolutionary modifications at key residues likely modulate cavity geometry, polarity, and local dynamics around the di-iron center, thereby potentially enabling hydroxylase activity in *FAH12*.

### 2.4. Evolutionary Origin and Divergent Functional Trajectories of FAD2 and FAH12 in Plants

**Figure 3:**
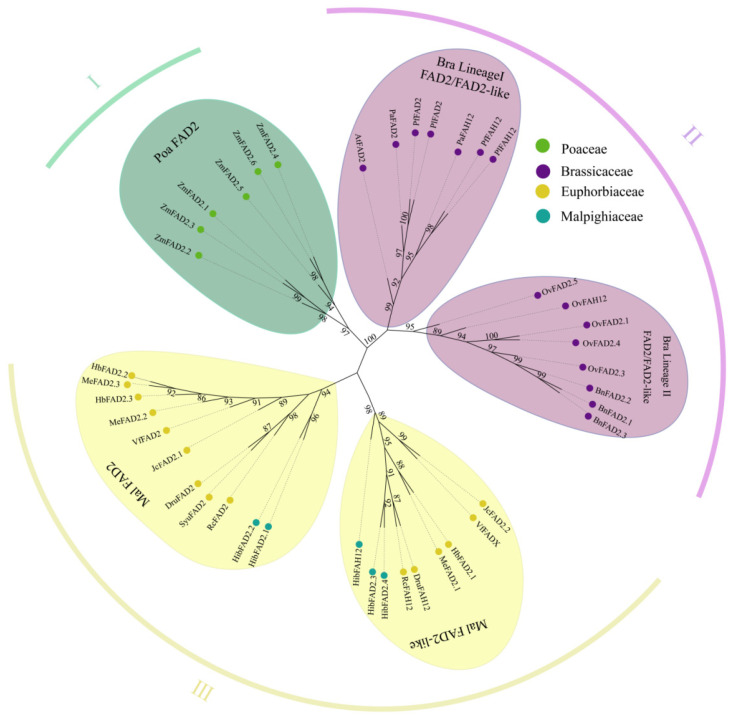
Phylogeny and evolution of the fatty acid desaturase (*FAD2*) family. A maximum-likelihood tree of FAD2 and FAD2-like proteins from 15 species resolves three well-supported clades. The ML tree was constructed using Jones–Taylor–Thornton (JTT) model, and bootstrap support values were calculated from 1000 replicates. Bootstrap values (≥50%) are shown at key nodes. Group I (green): *Z. mays* (Poaceae) desaturases only representing basal and conserved lineage, Group II (purple): Brassicaceae sequences splitting into *FAD2* desaturases and *FAD2*-like hydroxylases (*FAH12*), marking one origin of hydroxylase activity, Group III (yellow): Malpighiales sequences again separating into *FAD2* and *FAD2*-like hydroxylases, blue dots distinguish Malpighiaceae from Euphorbiaceae.

**Figure 4:**
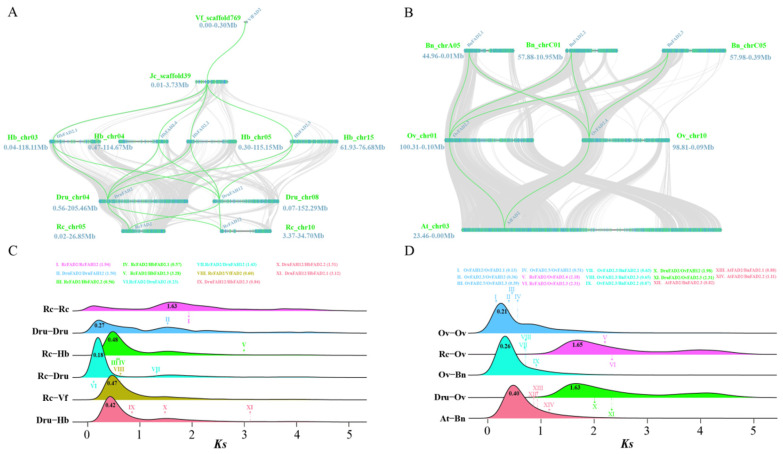
Macrosynteny and molecular evolutionary patterns of fatty acid desaturase (*FAD2*) and fatty acid hydroxylase 12 (*FAH12*) genes. (**A**) Collinearity relationships of *FAD2* and *FAH12* genes among five Euphorbiaceae species, including *Ricinus communis* (Rc), *Discocleidion rufescens* (Dru), *Hevea brasiliensis* (Hb), *Jatropha curcas* (Jc), and *Vernicia fordii* (Vf). (**B**) Synteny analysis of *FAD2* and *FAH12* genes among three Brassicaceae species, *Arabidopsis thaliana* (At), *Orychophragmus violaceus* (Ov), and *Brassica napus* (Bn). (**C**) Distribution of *synonymous substitution rate* (*Ks*) values for homologous gene pairs within and between Rc and Euphorbiaceae species (Dru, Hb, and Vf). (**D**) Distribution of *Ks* values for *FAD2* and *FAH12* homologs across Rc, Dru, and Brassicaceae species (Ov, Bn, and At). Grey lines in the background denote genome-wide collinear blocks, while green lines reflect the syntenic relationships between *FAD2* and *FAH12* genes. Colored peaks indicate duplication/speciation events, corresponding to Roman numerals and gene pairs listed above.

To trace the evolutionary origin of *FAH12* from ancestral *FAD2* genes, we reconstructed a comprehensive phylogeny of desaturase and hydroxylase homologs across representative monocots and dicots. The resulting tree resolved three major clades (Figure 3). Group I consisted primarily of monocot desaturases (e.g., *Z. mays FAD2*), representing a basal lineage. Group II comprised six Brassicaceae species, including Camelineae (*A. thaliana*), Physarieae (*P. lindheimeri*, *P. fendleri*, and *P. auriculata*), and Brassiceae (*B. napus* and *O. violaceus*) [28,29,30]. Group III encompassed seven Euphorbiaceae species and one representative of Malpighiaceae (*H. benghalensis*). Within each group, paralogs from the same species clustered together, indicating strong lineage-specific conservation. In Brassicaceae (Group II), two well-supported subclades were evident: one containing *B. napus* and *O. violaceus*, and another comprising *A. thaliana* and *Physarieae* species (Figure 3). In Euphorbiaceae (Group III), sequences were clearly separated into *FAD2*-type and *FAD2*-like branches. The *FAD2*-like branch included *FAH12* and its orthologs (e.g., *VfFADX*), as well as previously characterized hydroxylases such as *RcFAH12*, *DruFAH12*, *HibFAD2.3*, and *HibFAD2.4*. Importantly, *FAH12* homologs did not form a single monophyletic clade but instead grouped within lineage-specific *FAD2* related branches. This topology suggests that hydroxylase activity may have arisen independently from ancestral desaturases in multiple plant lineages, consistent with recurrent independent neofunctionalization (Figure 3). Furthermore, both *FAD2* and *FAD2*-like clades exhibited clear differentiation between Euphorbiaceae and Malpighiaceae (Figure 3), supporting lineage-specific evolutionary trajectories. Collectively, these phylogenetic patterns suggest that *FAH12* likely originated from ancestral *FAD2* genes through duplication events, followed by independent neofunctionalization in distinct taxonomic groups. To further investigate the evolutionary origin of *FAH12*, we performed intragenomic synteny analysis in *R. communis* and *D. rufescens*, revealing conserved collinearity between *FAD2* and its hydroxylase homolog *FAH12* within both genomes (Figure S9). Extending this analysis to five Euphorbiaceae species (*R. communis*, *D. rufescens*, *H. brasiliensis*, *J. curcas*, and *V. fordii*) showed strong syntenic conservation of *FAD2* loci across all species, whereas synteny of *FAH12* was only retained in a subset of species, including *R. communis*, *D. rufescens*, and *H. brasiliensis* (Figure 4A). These patterns suggest that the divergence between *FAD2* and *FAH12* likely predates the diversification of Euphorbiaceae species. In Brassicaceae, including *A. thaliana* (a Camelineae member) and *B. napus* and *O. violaceus* (two Brassiceae members), only *FAD2* genes exhibited strong interspecies synteny. Notably, *AtFAD2* (*A. thaliana*) was syntenic with *OvFAD2.3* and *OvFAD2.4* (*O. violaceus*) (Figure S9), and intragenomic comparisons further revealed collinearity between *OvFAD2.3* and *OvFAD2.4*. Integrating syntenic relationships with Ks values, we inferred that tandem duplication of *OvFAD2.4* and *OvFAH12* may have given rise to *OvFAD2.1* and *OvFAD2.5*, respectively (Figure 4A). These observations indicate that the origin of *OvFAH12* in *O. violaceus* likely followed a distinct evolutionary trajectory compared with Euphorbiaceae species. Together, the results support the widely accepted model that *FAH12*-type hydroxylases evolved from ancestral desaturases, while further suggesting that such transitions occurred independently in different plant lineages.

*Ks* and nonsynonymous-to-synonymous substitution ratios (*Ka*/*Ks*) were calculated for orthologous and paralogous gene pairs in Euphorbiaceae and Brassicaceae species. As shown in Figure 4C,D, *Ks* distributions indicated that both *FAD2* and *FAH12* lineages accumulated substitutions at rates higher than the genome-wide background, suggesting relatively accelerated evolutionary divergence. *Ks* values among *FAD2* orthologs were generally low (<0.9), consistent with recent divergence events. For example, *Ks* values within Euphorbiaceae were 0.23 (*RcFAD2*/*DruFAD2*), 0.56 (*RcFAD2*/*HbFAD2.2*), and 0.60 (*RcFAD2*/*VfFAD2*), respectively (Figure 4C). In Brassicaceae, *AtFAD2*/*BnFAD2* pairs exhibited *Ks* values of 0.82–1.11, while *OvFAD2.4*/*BnFAD2* pairs ranged from 0.63 to 0.87 (Figure 4D). These findings confirmed that *FAD2* paralogs were relatively recent products of duplication and speciation events. In contrast, *Ks* values between *FAD2* and *FAH12* paralogs were consistently higher (all > 1.4), including 1.94 (*RcFAD2*/*RcFAH12*), 1.50 (*DruFAD2*/*DruFAH12*), 1.43 (*RcFAD2*/*DruFAH12*), 1.51 (*DruFAH12*/*HbFAD2.2*), 2.18 (*RcFAD2*/*OvFAD2.4*), and 1.98 (*DruFAD2*/*OvFAH12*) (Figure 4C,D). These elevated values suggest that the divergence between *FAD2* and *FAH12* likely predates most lineage-specific genome duplication events, potentially arising from ancient ancestral duplications. Meanwhile, *Ka*/*Ks* ratios for nearly all gene pairs were <1 (Table S5), indicating strong purifying selection acting to preserve core catalytic features despite sequence divergence. Collectively, these results support a model in which *FAH12* originated from ancient *FAD2* duplication events, followed by prolonged neofunctionalization involving modifications to the substrate-binding environment that favor hydroxylation over desaturation. This process generated two functionally distinct yet structurally conserved enzyme lineages: (i) canonical Δ12-fatty acid desaturases catalyzing double-bond formation, and (ii) *FAH12*-type hydroxylases mediating oxygen insertion at the corresponding substrate position. Overall, this evolutionary framework highlights how gene duplication, lineage-specific divergence, and fine-scale structural tuning collectively contribute to the diversification of lipid-modifying enzymes in plants.

### 2.5. Expression Patterns and Regulatory Network of FAH12 and FAD2 in Castor

**Figure 5:**
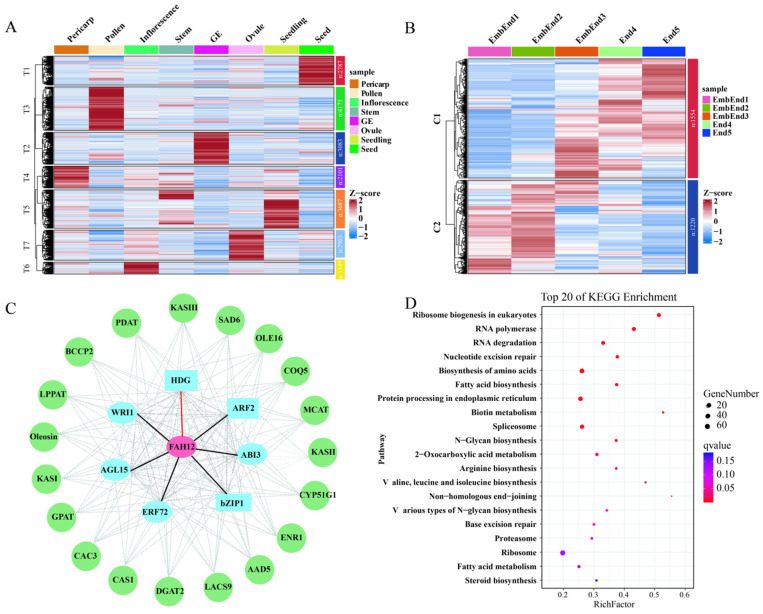
Spatiotemporal expression patterns and transcriptional network of *RcFAH12* and *RcFAD2* in castor bean. (**A**) Hierarchical clustering of gene expression profiles across diverse tissues. (**B**) Hierarchical clustering of gene expression profiles across different seed developmental stages, EmbEnd (mixed embryo and endosperm), End (endosperm), and Emb (embryo). The Z-score of gene expression levels has been applied for normalization. (**C**) Co-expression network of *RcFAH12* inferred using the gene-regulatory network reconstruction (GENIE3) algorithm. The network highlights putative regulatory relationships, with the first layer consisting of candidate transcription factors (TFs) and the second layer comprising downstream lipid metabolism genes. In the network, transcription factors are represented as blue ellipses, while lipid metabolism genes are shown as green circles. (**D**) KEGG pathway enrichment analysis of C1 genes.

To characterize the tissue-specific expression patterns of *FAH12* and *FAD2* in castor, transcriptomic datasets were collected from multiple tissues, including seedling, stem, pollen, inflorescence, ovule, pericarp, developing seed, and germinated endosperm (GE). A total of 19,885 genes with FPKM values greater than 0.1 in at least one sample were retained and subsequently classified into seven major tissue-associated clusters (Figure 5). Among these, 2787 genes were enriched in T1 (seed), 3083 in T2 (GE), 4175 in T3 (pollen), 2101 in T4 (pericarp), 3687 in T5 (stem and seedling), 1149 in T6 (inflorescence), and 2903 in T7 (ovule). Notably, *RcFAH12* was assigned to the seed-specified T1 cluster, whereas *RcFAD2* was grouped within the pollen-associated T3 cluster, indicating distinct spatial expression patterns. To further resolve the temporal dynamics of T1 cluster genes, five seed developmental stages at 5, 15, 25, 35, and 55 days after pollination (DAP) were analyzed and designated as S1–S5, respectively. These included embryo and endosperm tissues without seed coat from stages S1 to S3 (designated EmbEnd1–EmbEnd3) and developing endosperm from stages S4 to S5 (End4 and End5), each with three biological replicates (Figure 5A). K-means clustering of global expression profiles identified two major clusters: C1 (1554 genes, including *RcFAH12*), which was associated with the endosperm stage characterized by rapid fatty acid accumulation, and C2 (1220 genes), which was predominantly expressed during early seed development (Figure 5B). KEGG enrichment analysis revealed that C1 genes were significantly enriched in pathways related to RNA polymerase (ko03020), RNA degradation (ko03018), nucleotide excision repair (ko03420), fatty acid biosynthesis (ko00061), ER-specific protein processing (ko04141), and biotin metabolism (ko00780) (Figure 5D). Importantly, several key regulators of seed development and lipid metabolism—including *RcFAH12*, *RcLEC1*, *RcFUS3*, *RcWRI1*, *RcABI3*, *RcHDG*, *RcbZIP67*, and *RcbZIP1*—were co-enriched within the C1 cluster, highlighting a coordinated regulatory module associated with endosperm function. Regulatory network inference using the GENIE3 algorithm identified multiple transcription factors highly co-expressed with *RcFAH12*, including *RcERF72*, *RcARF2*, *RcbZIP1*, *RcHDG*, and *RcAGL15*, forming a primary regulatory layer (Figure 5C). A second layer consisted of lipid metabolism-related genes, such as *RcKASI*, *RcKASIII*, *RcSAD6*, *RcLACS9*, and *RcPDAT* (Figure 5C).

Promoter analysis of *FAH12* and *FAD2* homologs in castor, *O. violaceus*, and *D. rufescens* revealed diverse cis-regulatory elements that could be broadly categorized into three groups: (i) TF binding sites and core regulatory motifs, (ii) plant hormone–responsive elements, and (iii) development- and biosynthesis-related elements (Figure S10). TF-binding motifs included AP2/ERF, ATHB, HD-ZIP, bZIP, I-box, and RY/G box elements, suggesting multilayered transcriptional regulation of HFA biosynthesis. *ATHB* motifs were specifically detected in the promoters of *RcFAH12* and *DruFAH12*, while bZIP elements were present in *RcFAH12*, *RcFAD2*, *DruFAH12*, *OvFAD2.1*, and *OvFAD2.2*. HD-ZIP motifs were widely distributed across promoters of *RcFAH12*, *RcFAD2*, *DruFAH12*, *DruFAD2*, *OvFAD2.1*-*OvFAD2.4*, and *OvFAH12*. Notably, four RY/G box elements were identified in the *RcFAH12* promoter, indicating strong regulatory by seed-specific TFs (Figure S10). Hormone-responsive elements, including those responsive to auxin, cytokinin, and GA, further supported the involvement of hormone signaling in FAH12 regulation. GA-responsive elements (GARE) were detected in *RcFAH12*, *DruFAD2*, *DruFAH12*, and *OvFAD2.1*-*OvFAD2.4* promoters (Figure S10A). Additionally, developmental and biosynthesis-related motifs, such as endosperm-specific elements and storage protein-associated cis-elements, were identified, with four such elements present in the *RcFAH12* promoter (Figure S10A). To further identify transcription factor binding motifs significantly enriched in the promoters of *FAH12* and *FAD2* homologs, we performed Sequence Enrichment Analysis (SEA). The analysis identified a total of 6 significantly enriched motifs with E-values ≤ 0.05, corresponding to five transcription factor families: three motifs belonged to the bZIP family (TGA1A, TGA2_2, and bZIP910), while the remaining three represented the GATA (GATA14), NAC (ATAF1), and MYB (MYB10) families, respectively (Figure S10B). These results primarily reveal the cis-regulatory landscape governing *FAH12* and *FAD2* transcription, highlighting the central roles of bZIP TFs in regulating HFA biosynthesis. Collectively, our findings provide mechanistic insights into the transcriptional control of *FAH12*, pointing to a coordinated interplay between seed developmental programs, hormone signaling pathways, and metabolic regulation of HFA production.

### 2.6. Validation of RNA-Seq Data by qRT-PCR

**Figure 6:**
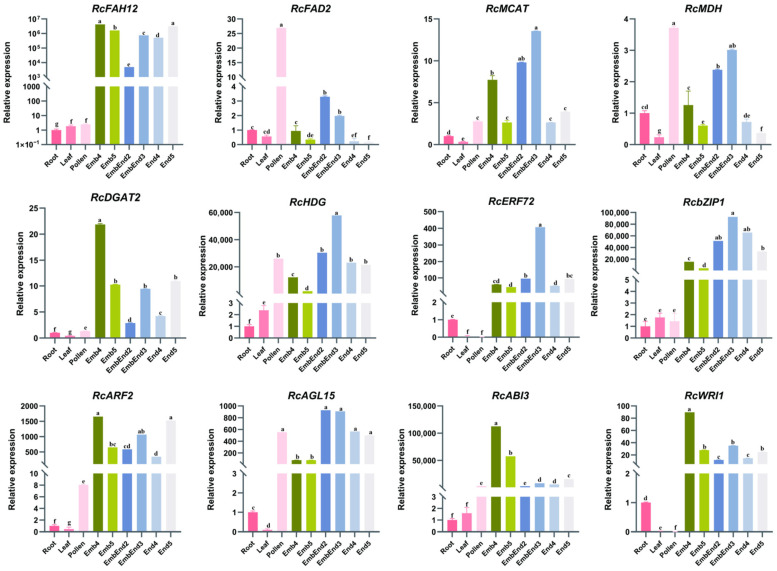
Validation of RNA-seq data by qRT-PCR analysis of selected genes in different tissues and seed developmental stages. Relative expression levels of 12 selected genes (*RcFAH12*, *RcFAD2*, *RcMCAT*, *RcMDH*, *RcDGAT2*, *RcHDG*, *RcERF72*, *RcbZIP1*, *RcARF2*, *RcAGL15*, *RcABI3*, and *RcWRI1*) were analyzed by qRT-PCR in root, leaf, pollen, and developing seeds. Seed samples included whole seeds at stages S2 and S3, as well as separately collected embryo (Emb) and endosperm (End) tissues at stages S4 and S5. Expression levels were normalized to the internal reference gene and are presented as relative expression values. Data represent mean ± standard deviation (SD) of three biological replicates. Different lowercase letters above the bars indicate statistically significant differences among samples (*p* < 0.05).

To validate the reliability of the transcriptomic data and further investigate the expression patterns of *RcFAH12* and *RcFAD2* across different tissues and seed developmental stages—particularly between embryo and endosperm tissues—quantitative real-time PCR (qRT-PCR) analysis was performed on 12 selected genes (Figure 6). These included *RcFAH12*, *RcFAD2*, and tricarboxylic acid (TCA) cycle-related gene *malate dehydrogenase* (*MDH*), fatty acid biosynthesis-related gene *malonyl-CoA:acyl carrier protein transacylase* (*MCAT*), TAG synthesis-related gene *diacylglycerol acyltransferase* (*DGAT2*), and several key transcription factors (*RcHDG*, *RcERF72*, *RcbZIP1*, *RcARF2*, *RcAGL15*, *RcABI3*, and *RcWRI1*) (Table S6).

Total RNA was extracted from root, leaf, pollen, and developing whole seeds, including whole seeds at S2 and S3, as well as separately isolated embryo and endosperm tissues at stages S4 and S5. The qRT-PCR results showed that *RcFAH12* and *RcDGAT2* exhibited low expression levels in vegetative tissues (root and leaf) and pollen, but were markedly upregulated during seed development (S2–S5), reaching peak expression at the late developmental stages (S4 and S5) in both embryo and endosperm (Figure 6). These expression patterns were highly consistent with the RNA-seq data. In contrast, *RcFAD2*, *RcMDH*, and *RcAGL15* displayed distinct expression profiles, with the highest expression observed in pollen, followed by relatively elevated expression during early to mid seed developmental stages (S2 and S3). Notably, *RcHDG*, *RcERF72*, and *RcbZIP1* exhibited expression trends similar to *RcFAH12*, supporting their potential involvement in the regulatory network controlling its expression (Figure 6). Additionally, *RcWRI1* and *RcABI3* showed coordinated expression dynamics during seed development, further supporting their roles in lipid biosynthesis regulation. Correlation relationship analysis revealed that 8 of the 12 selected genes exhibited significant positive correlations (R > 0.66, *p* < 0.05) between the RNA-seq and qRT-PCR results across different tissues and seed developmental stages (Figure S11). These results further confirmed the reliability and consistency of the RNA-seq data and reinforced the conclusion that *RcFAH12* is specifically activated during endosperm development.

## 3. Discussion

In this study, we integrated structural modeling, comparative evolutionary analyses, and transcriptomic profiling, together with qRT-PCR validation, to systematically dissect the molecular basis underlying the functional divergence between *RcFAH12* and *RcFAD2*. Although these enzymes share a common origin as ER-localized fatty acid desaturases, they catalyze distinct biochemical reactions on oleic acid (18:1), namely Δ12-desaturation and C12-hydroxylation. Our integrative analyses demonstrate that the emergence of *FAH12* activity cannot be attributed to a single structural or genetic change; rather, it results from the coordinated evolution of catalytic microenvironments, lineage-specific sequence divergence, and extensive regulatory reprogramming. Importantly, our findings extend beyond single-species observations by providing a cross-species framework, revealing that FAH12 evolution is characterized by repeated independent origins coupled with transcriptional specialization. This perspective refines the current understanding of hydroxy fatty acid (HFA) biosynthesis and helps explain why previous heterologous expression studies showed that *RcFAH12* alone is insufficient to achieve high HFA accumulation without appropriate regulatory context [31].

### 3.1. Structural Divergence and the Mechanistic Basis of Catalytic Switching

Comparative sequence and structural analyses across multiple plant species revealed that FAD2 proteins are highly conserved, with phylogenetic relationships mirroring species evolution, which is consistent with their essential role in membrane lipid desaturation. In contrast, FAH12 proteins display substantially greater sequence variability, particularly at residues previously implicated in catalytic divergence [20]. Notably, no single amino acid substitution universally distinguishes *FAH12* from *FAD2* across all plant lineages. Instead, our results support a model in which functional divergence arises from coordinated, multi-residue changes, rather than from a single “switch” mutation. This observation is consistent with earlier mutagenesis studies and is further extended to a cross-species evolutionary framework in the present work [20].

Despite these differences, both *FAH12* and *FAD2* retain the conserved three-histidine motif that coordinates the di-iron catalytic center, thereby maintaining the ancestral desaturase scaffold [32]. Structural modeling and substrate docking further revealed that the transition from desaturation to hydroxylation can be explained by subtle but coordinated modifications of the substrate-binding pocket, rather than large-scale conformational changes (Figure 2). Such structural conservation provides a shared biochemical template upon which subtler modifications could act, explaining the inadequacy of simple loss-of-function mutations in generating new chemistry in several earlier studies [31]. Specifically, amino acid substitutions flanking the substrate channel differ between *RcFAH12* (S, G, I, F, V, A, V) and *RcFAD2* (A, A, T, Y, A, S, M). These substitutions collectively alter side-chain polarity, hydrophobicity, and steric constraints (e.g., G/A vs. T/M, F vs. Y) (Figure 2), which are predicted to reshape local structural packing and hydrogen-bonding networks [11]. Specifically, these substitutions may (i) enhance local conformational flexibility, (ii) weaken hydrogen-bonding constraints, and (iii) enlarge the effective volume of the catalytic pocket. These combined modifications constitute key determinants governing acyl chain accommodation, as documented in the phytopathogenic fungus *Claviceps purpurea* [11]. As a result, *RcFAH12* forms a more permissive and dynamically adjustable substrate tunnel, facilitating optimal positioning of the oleoyl C12 carbon toward the reactive di-iron–oxo intermediate (Figure 2). In contrast, *RcFAD2* maintains a narrower and more hydrophobic channel that constrains substrate orientation, favoring hydrogen abstraction and double bond formation (Figure 2). Therefore, the catalytic outcome is determined not by the catalytic center itself, but by the spatial presentation of the substrate within the catalytic pocket. Taken together, these findings support a microenvironment-driven mechanism of enzymatic neofunctionalization, in which cumulative physicochemical alterations reshape the catalytic pocket to favor hydroxylation. This model also explains why no universally conserved diagnostic residue exists across *FAH12* enzymes: functional conversion likely requires coordinated tuning of multiple neighboring residues rather than a single substitution event.

### 3.2. Recurrent Independent Origins of FAH12 Through Lineage-Specific Neofunctionalization

Our phylogenetic and synteny analyses provide strong evidence that *FAH12* did not arise from a single ancestral event but instead originated independently in multiple plant lineages through duplication of ancestral *FAD2* genes followed by neofunctionalization (Figure 3). In Euphorbiaceae, conserved *FAD2*–*FAH12* gene pairs and distinct *FAH12*-specific clades indicate an early duplication event followed by lineage-specific divergence. These findings parallel the convergent evolution of oleate hydroxylases previously documented in Bacillales [11]. In contrast, within Brassicaceae, *FAH12* homologs cluster more closely with species-specific *FAD2* genes, suggesting a more recent and independent origin. Together, these contrasting evolutionary patterns strongly support a model of recurrent independent neofunctionalization of hydroxylase activity.

Molecular evolutionary analyses further reinforce this model. Although *Ka*/*Ks* ratios below 1 indicate strong purifying selection maintaining the core desaturase fold, elevated *Ks* values between *FAD2* and *FAH12* suggest ancient divergence events predating recent speciation (Figure 4). This combination of structural conservation and sequence divergence is characteristic of enzymes undergoing neofunctionalization while retaining essential catalytic frameworks, as observed in RA-producing species such as castor, *D. rufescens*, and *O. violaceus*. Similar selective patterns have been reported in other lipid-related gene families in *Sesamum indicum* [33]. Importantly, the absence of universally conserved hydroxylase-specific residues across lineages aligns with the structural findings described above, further supporting the notion that *FAH12* evolution follows lineage-specific evolutionary trajectories rather than a single conserved mutational pathway at the sequence level. Collectively, these results support a unifying evolutionary model in which *FAH12* repeatedly emerges from ancestral *FAD2* genes through gene duplication followed by lineage-specific accumulation of mutations that reshape catalytic microenvironments and confer hydroxylase activity.

### 3.3. Regulatory Rewiring Drives Functional Specialization of FAH12

In addition to structural and evolutionary divergence, our transcriptomic and qRT-PCR analyses reveal that regulatory specialization represents a critical dimension of *FAH12* functional evolution. Despite their shared ancestry, *RcFAH12* and *RcFAD2* exhibit strikingly distinct expression patterns (Figure 5). *RcFAH12* is highly and specifically expressed in developing endosperm during the phase of rapid oil accumulation, whereas *RcFAD2* displays broader expression patterns, including strong expression in pollen and early developmental stages. These differences indicate substantial transcriptional decoupling between the two genes.

Importantly, qRT-PCR validation confirmed that *RcFAH12* expression is strongly induced during mid-to-late seed development (S3–S5), consistent with the transcriptomic data, whereas *RcFAD2* follows a distinct temporal expression pattern. This experimental validation strengthens the reliability of RNA-seq–based inferences and further supports the conclusion that *RcFAH12* is specifically associated with endosperm-driven lipid accumulation. Regulatory network inference and promoter analyses further revealed that *RcFAH12* is embedded within a seed-specific transcriptional module. In particular, *RcHDG*, a HD-ZIP IV transcription factor, exhibited strong co-expression and highly consistent expression dynamics with *RcFAH12*, as verified by qRT-PCR (Figure 6). Cis-element analysis demonstrated that HD-ZIP binding motifs are present in the *RcFAH12* promoter but absent from the *RcFAD2* promoter, providing molecular evidence for their divergent regulatory architectures. Moreover, the identified HD-ZIP binding motif contains the canonical TAAA core sequence, a hallmark of HD-ZIP IV recognition sites, supporting the hypothesis that *RcHDG* may directly regulate *RcFAH12* transcription. Additional co-expressed transcription factors, including *RcWRI1* and *RcABI3*, further connect *RcFAH12* to the well-established seed oil biosynthesis regulatory network in castor. In addition to HD-ZIP IV factors, other transcription factor families, including bZIP, ERF, and MYB, are also predicted to participate in the transcriptional modulation of HFA biosynthesis. Accumulating evidence indicates that bZIP and MYB TFs serve as key regulators of fatty acid and specialized metabolite biosynthesis across diverse plant species [34]. Unlike the well-characterized embryo-centered regulatory systems in *Arabidopsis* [35,36], castor oil biosynthesis occurs predominantly in the endosperm. Our findings thus suggest the existence of a distinct endosperm-specific regulatory architecture, in which *RcHDG* may act as a key activator driving *RcFAH12* expression and subsequent hydroxy fatty acid accumulation.

Future studies integrating site-directed combinatorial mutagenesis, yeast one-hybrid assays, electrophoretic mobility shift assays (EMSA), and genome-editing approaches will be essential to experimentally validate the direct regulatory relationship between *RcHDG* and *RcFAH12*. From an applied perspective, coordinated engineering of both *FAH12* catalytic activity and its associated transcriptional network may represent a promising strategy for enhancing ricinoleic acid production in heterologous oilseed crops. Collectively, our findings provide not only mechanistic insights into *FAH12* evolution, but also a theoretical foundation for future metabolic engineering and synthetic biology efforts aimed at the sustainable industrial production of hydroxy fatty acids.

## 4. Materials and Methods

### 4.1. Plant Materials and Sample Preparation

The castor cultivar ZB306 served was used in this study. Plants were grown under controlled greenhouse conditions at Southwest Forestry University (Kunming, Yunnan Province, China) from April to November 2020. Developing seeds were collected at five defined developmental stages—5, 15, 25, 35, and 55 days after pollination—designated as S1–S5, respectively. For each stage, three independent biological replicates were harvested. Seeds were dissected into outer seed coat, inner seed coat, endosperm, and embryo. All samples were immediately frozen in liquid nitrogen and stored at −80 °C until further analyses.

### 4.2. Identification of FAH12 and FAD2 Genes

To investigate the evolutionary relationships of *FAH12* and *FAD2* genes, genomic datasets from 11 representative monocot and dicot species were analyzed, with particular emphasis on Euphorbiaceae and Brassicaceae lineages. The species included *R. communis* L., *D. rufescens*, *S. tuberculata*, *J. curcas* L., *M. esculenta* Crantz, *H. brasiliensis*, *V. fordii*, *O. violaceus*, *B. napus* L., *A. thaliana*, and *Z. mays*. Genomic resources were retrieved from public databases, including the National Genomics Data Center (NGDC, https://ngdc.cncb.ac.cn/gsa-human/browse/CRA008040, accessed on 14 August 2024) for *O. violaceus*, EupDB (http://eupdb.liu-lab.com/keywords_search/, accessed on 14 August 2024) for Euphorbiaceae species comprising *J. curcas* (v2.0), *M. esculenta* (v6.1), *H. brasiliensis* (v1.1), and *V. fordii* (v1.0), TAIR (https://www.arabidopsis.org/, accessed on 14 August 2024) for *A. thaliana*, MaizeGDB (https://www.maizegdb.org/, accessed on 14 August 2024) for *Z. mays* [37]. Genome assemblies of *R. communis*, *D. rufescens*, and *S. tuberculata* were generated in our laboratory. Additionally, *FAH12* and *FAD2* sequences from *P. fendleri*, *P. lindheimeri*, and *Paysonia auriculata* were obtained from GeneBank (https://www.ncbi.nlm.nih.gov/genbank/, accessed on 14 August 2024) [9,28]. *RcFAH12* (28035.m000362) and *RcFAD2* (29613.m000358) were used as query sequences for homology searches [26]. Homologs were identified using BLASTP (E-value < 1 × 10^−5^), followed by manual filtering (identity > 50%, coverage > 70%) [38]. Hidden Markov Model (HMM) searches were conducted using PFAM domains PF11960 and PF00487 (E-value < 1 × 10^−10^) [39]. Candidate genes identified via BLASTP and HMMER (http://hmmer.org/, accessed on 20 August 2024) were compared employing the Venny platform (https://bioinfogp.cnb.csic.es/tools/venny/, accessed on 20 August 2024). Candidate genes were validated via SMART (http://smart.emblheidelberg.de/, accessed on 20 August 2024). Gene structures were visualized using GSDS2.0 (Gene Structure Display Server 2.0, https://gsds.gao-lab.org/) [40,41]. Phylogenetic trees, exon–intron organization, and motif architectures were jointly visualized using TBtools-II [38]. Protein physicochemical characteristics—including molecular mass, theoretical isoelectric point (pI), and amino acid length—were predicted using the ExPASy ProParam tool (https://web.expasy.org/protparam/, accessed on 24 August 2024). Subcellular localization was inferred using both Plant-mPLoc v2.0 (http://www.csbio.sjtu.edu.cn/bioinf/plant/, accessed on 27 August 2024) and DeepLoc 2.1 (https://services.healthtech.dtu.dk/, accessed on 27 August 2024) to improve prediction reliability [42,43].

### 4.3. Sequence Alignment, Structural Analysis, and Molecular Docking

Protein sequences of *FAH12* and *FAD2* were aligned using MAFFT under default parameters. Conserved functional motifs and structural domains were annotated via InterPro (https://www.ebi.ac.uk/interpro/, accessed on 4 October 2024) online database [44], and sequence alignment features together with conserved residue distribution were visualized using ESPript 3.0 (https://espript.ibcp.fr/ESPript/cgi-bin/ESPript.cgi, accessed on 4 October 2024) [45,46]. Homology modeling of FAH12 and FAD2 protein three-dimensional structures was performed using AlphaFold Server (https://deepmind.google/science/alphafold/alphafold-server/, accessed on 12 May 2026). To guarantee structural reliability, only predicted models with a TM-score > 0.7 and high pLDDT confidence were retained for subsequent structural analysis and pocket calculation. Active-site cavity and pocket volume were quantitatively calculated using the DoGSite3 algorithm embedded in the Proteins Plus Server (https://proteins.plus/, accessed on 11 October 2024). Molecular docking simulation was carried out using AutoDockTools 1.5.7 and Autodock Vina, with oleic acid (PubChem, CID: 445639) selected as the ligand molecule [47,48]. Key binding residues and molecular interaction patterns were further analyzed using Discovery Studio 2019, and structural diagrams were visualized and rendered with PyMOL [49,50].

### 4.4. Phylogenetic and Duplication Analysis

Sequence similarity was assessed through multiple sequence alignment using MAFFT with the auto strategy. Phylogenetic trees were constructed using the Maximum Likelihood (ML) method in MEGA (v0.10.5), with the Jones–Taylor–Thornton (JTT) model and 1000 bootstrap replicates to assess node support [51,52]. And the trees were visualized and annotated using iTOL (https://itol.embl.de/, accessed on 14 October 2024) [53]. Synteny analysis, *Ks* estimation, and duplication inference were performed using the WGDI (Whole Genome Duplication and Interspecies Divergence) toolkit based on the YN (Yang–Nielsen) model [54].

### 4.5. Transcriptome Sequencing and Expression Analysis

Total RNA was extracted from seven tissue samples and developing seeds (endosperm and embryo, or endosperm only) across five developmental stages (S1-S5) using the RNAprep pure Plant Kit (Tiangen Biotech, Beijing, China). RNA-seq libraries were constructed using the NEBNext Ultra™ II RNA Library Prep Kit (New England Biolabs, Ipswich, MA, USA) and sequenced on the Illumina NovaSeq 6000 platform. Clean reads were aligned to castor reference genome through HISAT2 v2.2.1 [55]. Gene expression levels were quantified as fragments per kilobase of transcript per million fragments mapped (FPKM) values using StringTie v2.2.0 [56]. Differentially expressed genes (DEGs) were identified using DESeq2 (|log2 Fold Change|> 2, *p*-value < 0.05) [57]. Hierarchical clustering of gene expression profiles was performed using the ClusterGVis package (https://github.com/junjunlab/ClusterGVis/, accessed on 14 February 2025). The optimal number of clusters was determined the getClusters function, followed by k-means clustering to classify gene expression patterns, which were visualized as heatmaps [58]. Gene co-expression regulatory networks were inferred using GENIE3 (v1.30.0) [59]. The resulting co-expression network was visualized in Cytoscape v3.9.1 [60].

To further identify the transcription factor binding sites and their conservation, promoter sequences (2 kb upstream regions) of *FAH12* and *FAD2* genes were extracted and analyzed for cis-regulatory elements via the PlantPAN 4.0 database (http://PlantPAN.itps.ncku.edu.tw/, accessed on 9 April 2025) [61], and the results were illustrated with TBtools [38]. In parallel, the same promoter sequences of *FAH12* and *FAD2* homologs were subjected to Sequence Enrichment Analysis (SEA, v5.5.9, https://meme-suite.org/meme/tools/sea, accessed on 10 April 2025) to detect significantly enriched cis-regulatory motifs. The target promoters were used as the primary dataset, and 1000 random promoter sequences from castor were included as a control background set. SEA was run on both DNA strands to capture motifs regardless of orientation. A 2nd-order Markov background model was built from the control sequences to account for inherent nucleotide composition bias. Control sequences were generated by shuffling while preserving 3-mer frequencies, and Fisher’s exact test was used to evaluate differential motif enrichment. Motifs with an enrichment E-value ≤ 0.05 were considered statistically significant. Identified motifs were annotated against the JASPAR2026 CORE plant non-redundant database, and corresponding sequence logos and position weight matrices (PWMs) were retrieved to characterize their conserved sequence features.

### 4.6. qRT-PCR Analysis

Total RNA was extracted from leaf, pollen, and developing whole seeds at stage S2, developing embryo and endosperm tissues at stages S4–S5. First-strand cDNA was synthesized using a reverse transcription kit following the manufacturer’s instructions. qRT-PCR was conducted using SYBR Green chemistry on a real-time PCR system. *RcACTIN* was used as the internal reference gene. Relative expression levels were calculated using the 2^−ΔΔCt^ method [62]. Each experiment included three independent biological replicates, and each biological replicate was analyzed with three technical repeats. Relative expression levels were presented as mean ± standard deviation (SD). Statistical analyses were conducted in R (v4.3.1) using the agricolae (https://CRAN.R-project.org/package=agricolae/, accessed on 5 May 2026) and ggpubr (https://CRAN.R-project.org/package=ggpubr/, accessed on 6 May 2026) packages. Differences among multiple groups were assessed using one-way analysis of variance (ANOVA), followed by least significant difference (LSD) multiple comparison tests. A *p*-value < 0.05 was considered statistically significant. Pearson’s correlation analysis was performed to evaluate relationships between RNA-seq and qRT-PCR results, and correlation coefficients were visualized using ggpubr package.

## 5. Conclusions

In summary, this study provides a multi-layered dissection of the divergence between *RcFAH12* and its ancestral *RcFAD2* through the integration of structural, evolutionary, and transcriptomic analyses. We demonstrate that *RcFAH12* has acquired subtle yet functionally decisive modifications in its substrate-binding pocket, enabling the shift from Δ12-desaturation to hydroxylation. Evolutionarily, *RcFAH12* originated through gene duplication followed by lineage-specific neofunctionalization under sustained purifying selection. At the transcriptomic level, *RcFAH12* underwent transcriptional rewiring, resulting in highly specialized expression in the developing mid-late endosperm. Importantly, the incorporation of qRT-PCR validation strengthens the robustness of the transcriptomic findings and provides experimental support for the proposed regulatory divergence. Collectively, these findings establish *RcFAH12* as a key evolutionary innovation in plant lipid metabolism and provide a unified framework linking structural adaptation, evolutionary origin, and regulatory specialization. From an applied perspective, the identification of critical amino acid residues, transcription factors, and cis-regulatory elements offers valuable targets for metabolic engineering. In particular, the lineage-specific sequence features and regulatory characteristics identified in this study may guide the rational design or optimization of FAH12-mediated pathways in heterologous oilseed systems to improve hydroxy fatty acid accumulation. These insights pave the way for introducing hydroxylase activity into heterologous oilseed crops, thereby enabling sustainable production of hydroxy fatty acids and reducing reliance on castor cultivation.

## Data Availability

The RNA-seq data generated in this study have been deposited in NCBI under the BioProject accessions PRJNA787114 (https://www.ncbi.nlm.nih.gov/sra/?term=PRJNA787114, accessed on 12 May 2026).

## Supplementary Materials

The following supporting information can be downloaded at: https://www.mdpi.com/article/10.3390/plants15101544/s1, Figure S1: Identification of the *FAD* gene family in castor bean; Figure S2: Evolutionary relationships of *FAD* subfamilies between *R. communis* and *Arabidopsis thaliana*; Figure S3: Phylogenetic relationship, conserved domain, and gene structure of *FAD* family members across 13 plant species; Figure S4: Multiple sequence alignment of *FAD2* and *FAH12* protein family members; Figure S5: Pairwise alignment of the full-length amino-acid sequences of *RcFAD2* and *RcFAH12* from *R. communis*; Figure S6: Multiple sequence alignment of *DruFAH12*, *RcFAH12* and *PlFAH12* proteins; Figure S7: Model confidence assessment of *RcFAD2* and *RcFAH12*; Figure S8: Predicted binding pocket volume of individual *FAD2* and *FAH12* homologs; Figure S9: Synteny analysis of the genomic regions harboring *FAD2* and *FAH12* genes; Figure S10: Analysis of cis-regulatory elements in the promoters of *FAD2* and *FAH12* genes; Figure S11: Correlation between qRT-PCR and RNA-seq expression measurements for selected genes in *R. communis*. Table S1: Identification of *FAD2* genes using BLASTP in castor bean; Table S2: Identification of *FAD2* genes using HMMER in castor bean; Table S3: Detailed information of candidate *FAD* genes; Table S4: *Ka* and *Ks* analysis of *FAD2* and *FAH12* genes; Table S5: Predicted binding pocket properties of *FAD2* and *FAH12* homologs; Table S6: Gene-specific primers used for qRT-PCR.
