# Supplementary material for: Structural, Evolutionary, and Regulatory Divergence of FAH12 from FAD2 Reveals Recurrent Independent Neofunctionalization Underlying Ricinoleic Acid Biosynthesis in Ricinus communis
Source: Plants (Basel). 2026 May 19;15(10):1544. doi: 10.3390/plants15101544 (PMC13211250; doi:10.3390/plants15101544)
Supplement: Supplementary file 1 [file plants-15-01544-s001.zip › plants-4289813-supplementary.pdf]

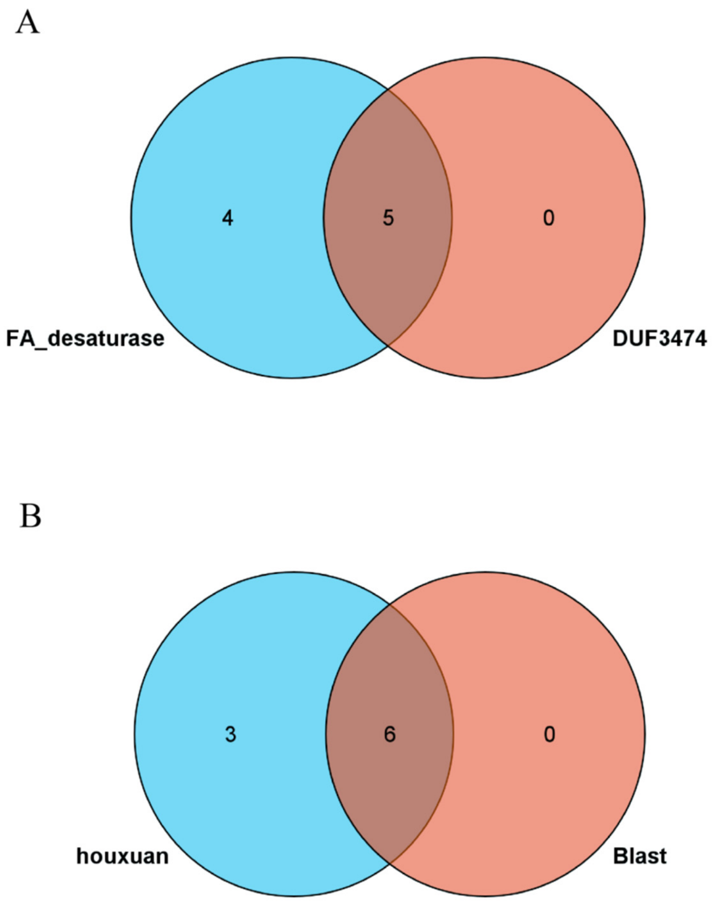

Fig. S1. Identification of the *FAD* gene family in castor bean. (A) Number of candidate genes identified by initial BLASTP search. (B) Number of candidate genes identified by HMMER search using PFAM domains (PF00487, PF11960). The intersection in the Venn diagram shows the core *FAD* gene family members identified by both methods and selected for subsequent phylogenetic analysis.

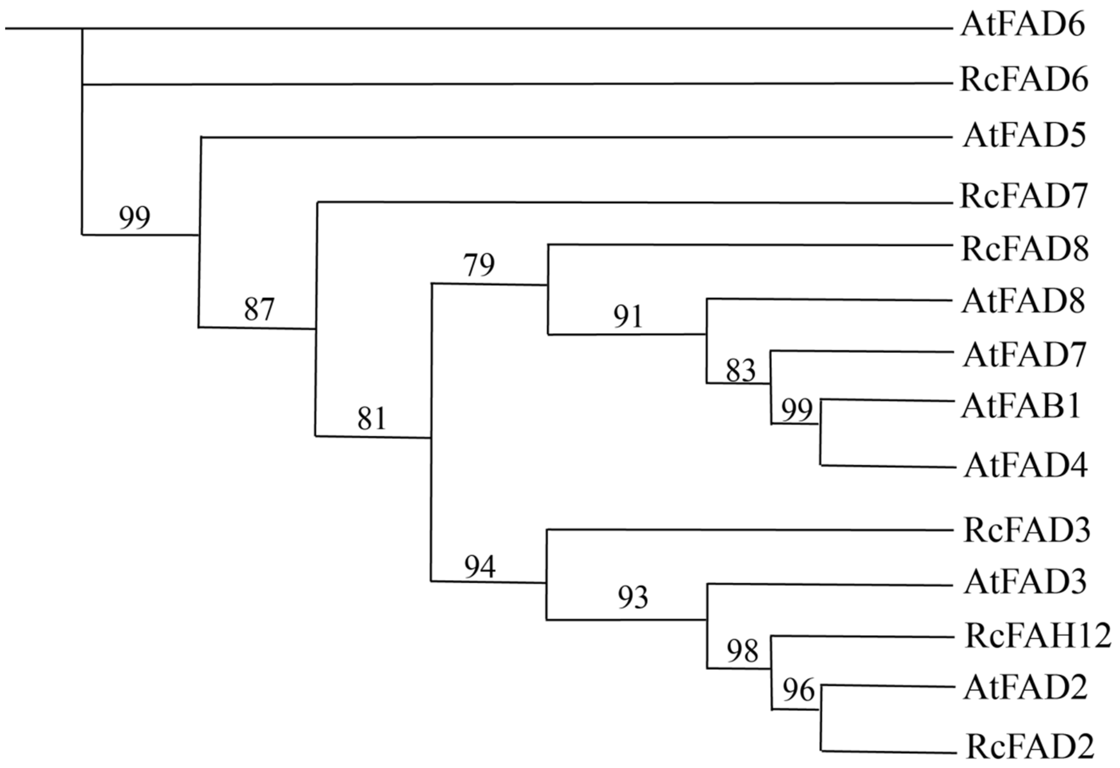

Fig. S2. Evolutionary relationships of *FAD* subfamilies between *Ricinus communis* and *Arabidopsis thaliana*. The maximum-likelihood tree illustrates the evolutionary relationships between *FAD* homologs from *R. communis* (Rc) and *A. thaliana* (At). Orthologous gene pairs (e.g., *FAD7*, *FAD8*) cluster together, confirming conserved evolutionary patterns within these *FAD* subfamilies.

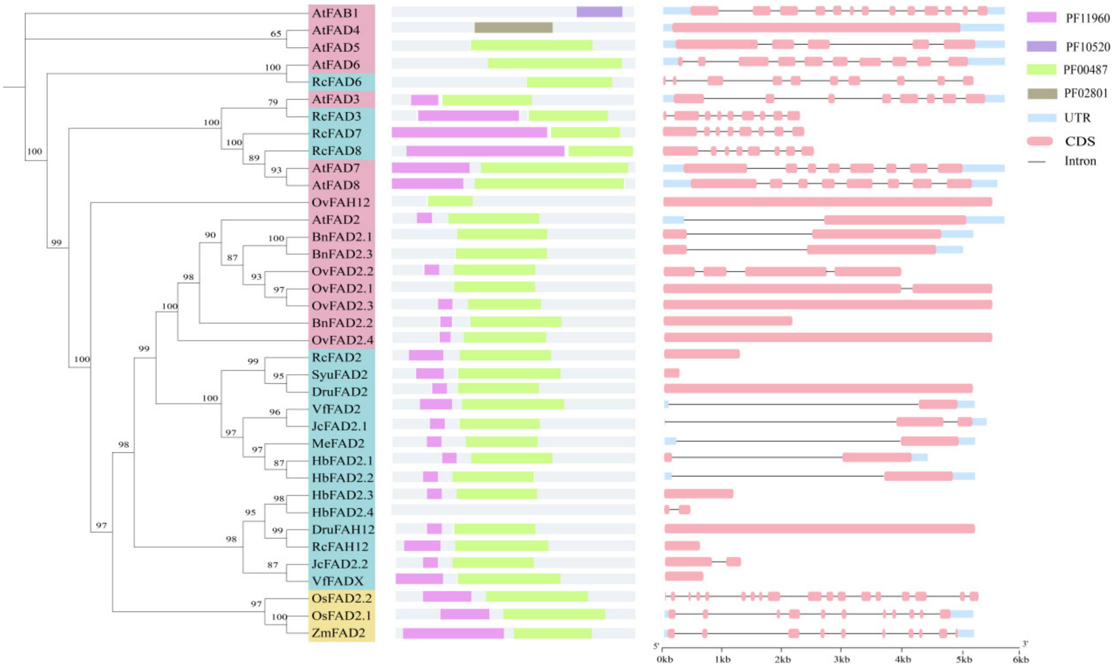

Fig. S3. Phylogenetic relationship, conserved domain, and gene structure of *FAD* family members across 13 plant species. Conserved protein domains of PF11960 (DUF3474 domain), PF00487 (FA_desaturase domain), PF10520 (FA_hydroxylase), PF02801 (Cyb5 domain) were represented as mauve, green, violet, and gray, respectively. The exon and intron were represented as red squares and black line, respectively.

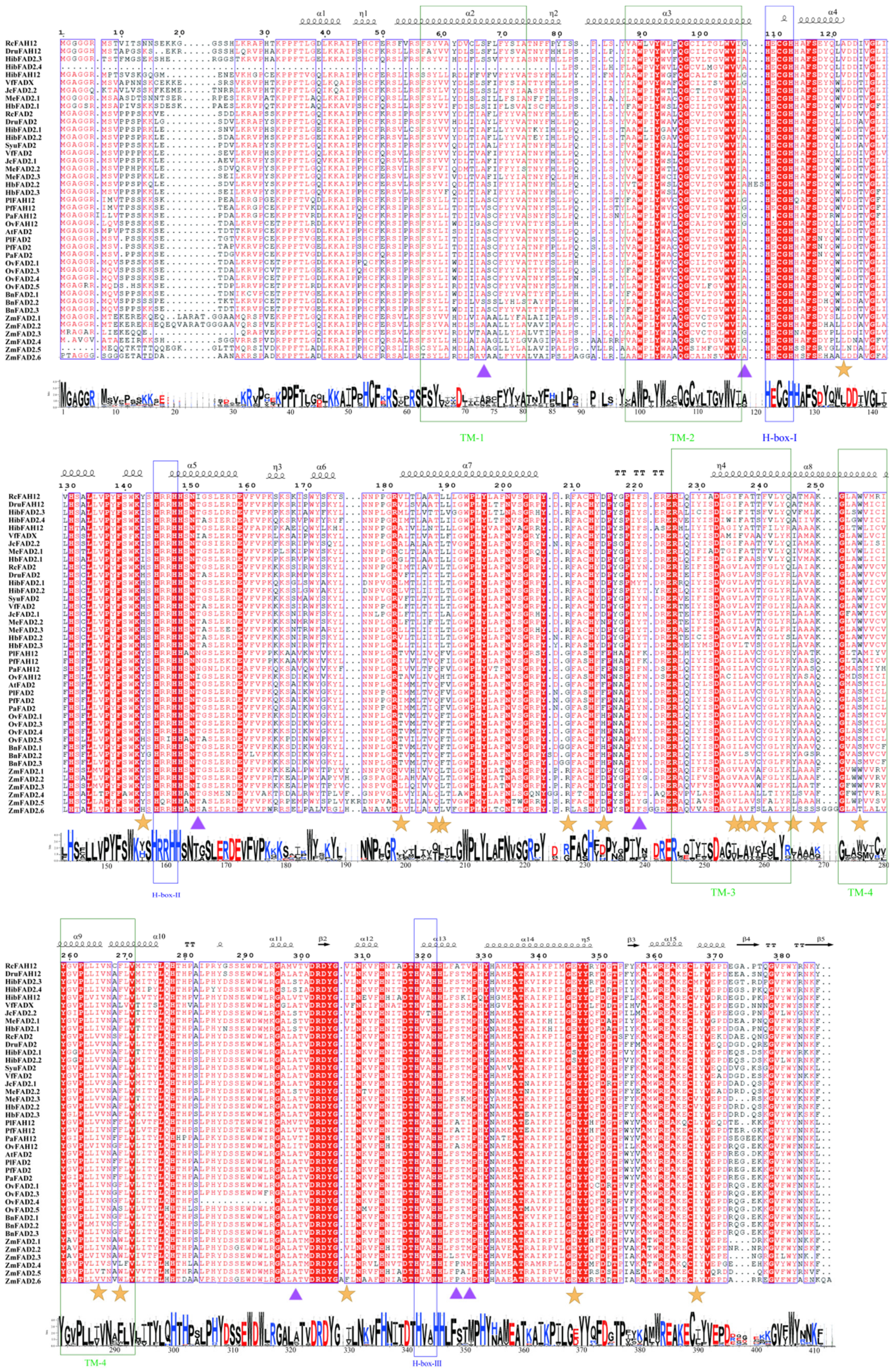

Fig. S4. Multiple sequence alignment of *FAD2* and *FAH12* protein family members. The sequence conservation score (in bits) is plotted above the alignment,

with taller peaks indicating higher conservation. Key structural and functional elements are annotated: the three highly conserved histidine boxes (H-box I, II, and III), which coordinate the essential di-iron catalytic center, are highlighted in blue. The four predicted transmembrane helices (TM 1-4) are indicated with green boxes. Beyond the conserved catalytic motifs, multiple amino acid positions (including the previously described seven diagnostic residues and the newly identified sites in this study) exhibit function-specific (hydroxylation vs. desaturation) and/or lineage-specific substitutions, providing the molecular basis for their functional divergence.

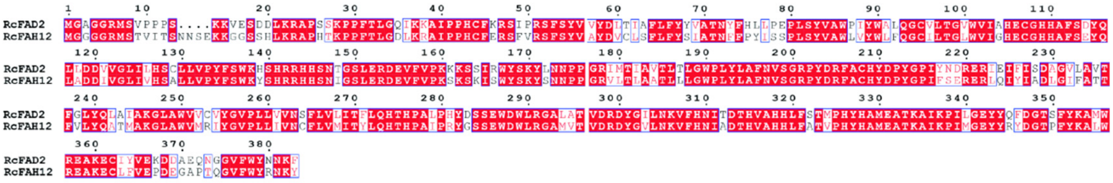

	RcFAD2	RcFAH1
RcFAD2	100.0	73.9
RcFAH12	73.9	100.0

RcFAD2 vs RcFAH12 : 73.89%

Fig. S5. Pairwise alignment of the full-length amino-acid sequences of *RcFAD2* and *RcFAH12* from Ricinus communis. Identical residues are shown with a red background, similar residues in black, and dashes denote gaps introduced for optimal alignment. The overall sequence identity between *RcFAD2* and *RcFAH12* is 73.89%.

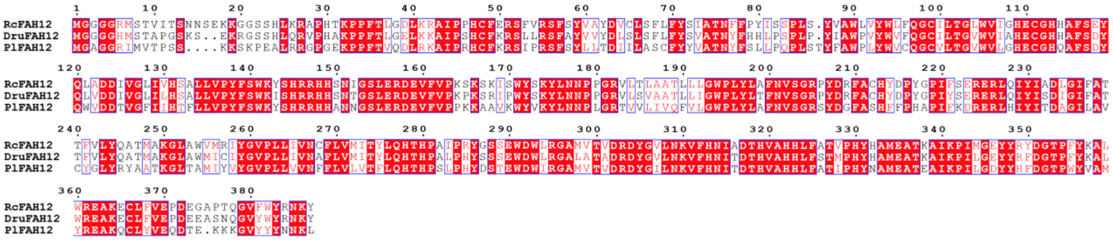

	RcFAH12	DruFAH12	PlFAH12
RcFAH12	100.0	82.6	63.6
DruFAH12	82.6	100.0	65.2
PlFAH12	63.6	65.2	100.0

RcFAH12 vs DruFAH12 : 82.60%

RcFAH12 vs PlFAH12 : 63.61%

DruFAH12 vs PlFAH12 : 65.18%

Fig. S6. Multiple sequence alignment of *DruFAH12*, *RcFAH12* and *PlFAH12* proteins. Identical residues are highlighted in red, similar residues in black, and gaps introduced for alignment are indicated by dashes. The homology of *RcFAH12* and *DruFAH12* is 82.60%, but the homology with *PlFAH12* is only 63.61%, and the homology between *DruFAH12* and *PlFAH12* is 65.18%.

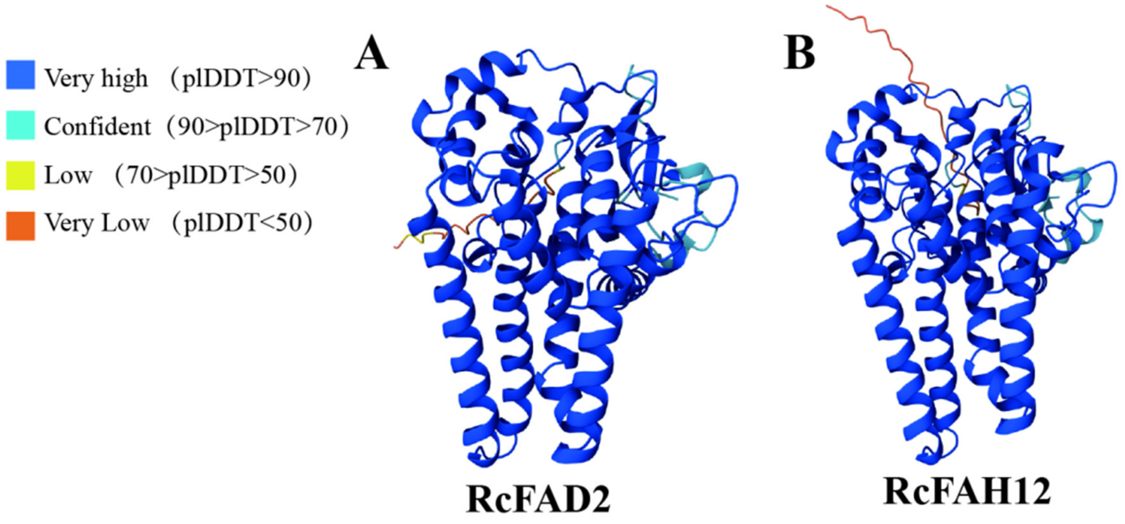

Fig. S7. Model confidence assessment of *RcFAD2* and *RcFAH12*. (A) *RcFAD2*: over 90% of residues exhibit very high confidence (pLDDT > 90). (B) *RcFAH12*: over 90% of residues exhibit very high confidence (pLDDT > 90). (pLDDT > 90 indicates that the predicted structure is highly reliable).

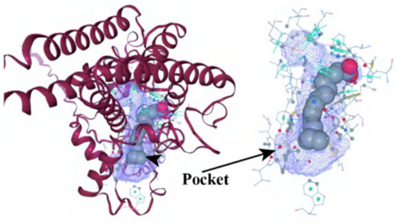

OvFAD2.5 (803.33 Å³)

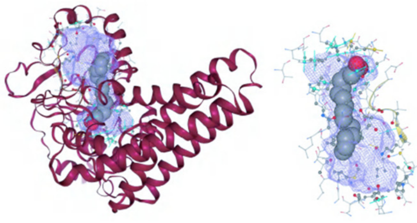

OvFAD2.4 (998.4 Å³)

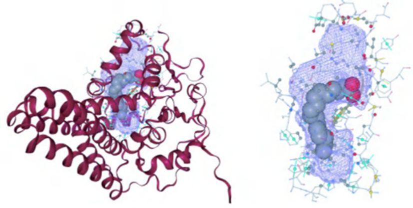

OvFAH12 (1059.33 Å³)

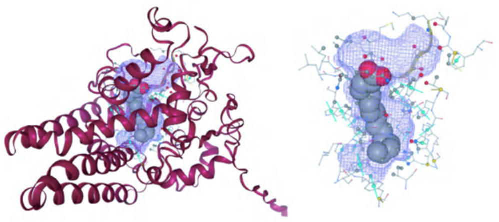

OvFAD2.3 (930.82Å³)

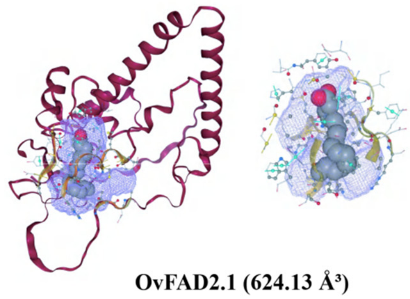

OvFAD2.1 (624.13 Å³)

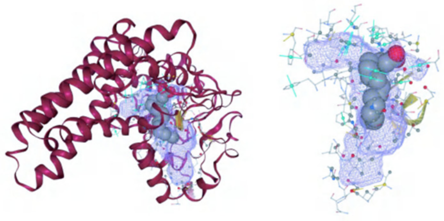

PlFAH12 (814.08 Å³)

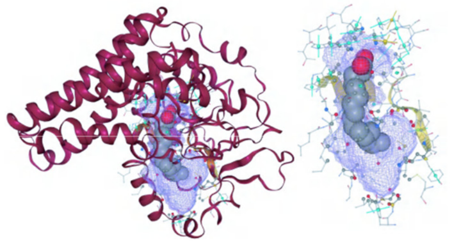

DruFAD2 (500.74 Å³)

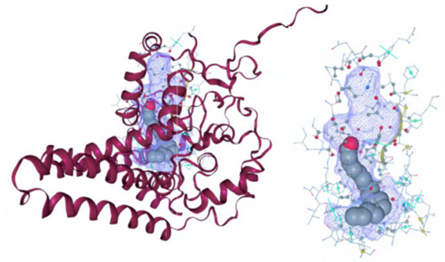

DruFAH12 (735.74 Å³)

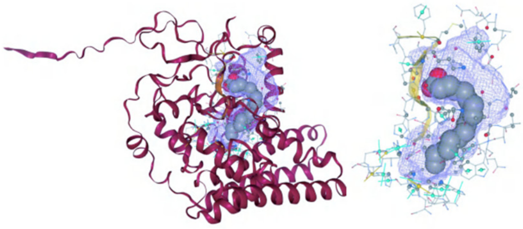

HibFAD2.3 (338.94 Å³)

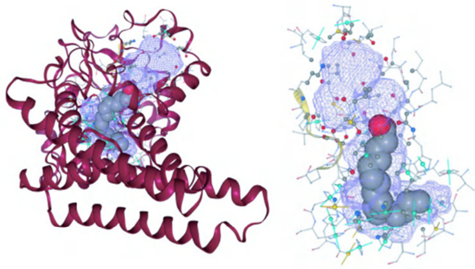

HibFAD2.2 (651.78 Å³)

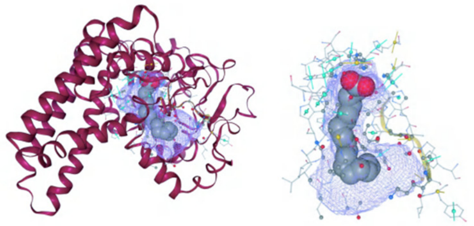

HibFAD2.1 (728.58 Å³)

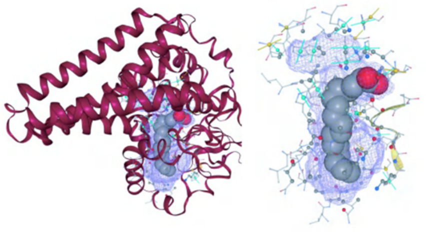

HibFAH12 (826.88 Å³)

Fig. S8. Docking-derived substrate-bound conformations of individual *FAD2* and *FAH12* homologs. For each protein, the three conserved histidine boxes (I–III) are shown in red and the predicted substrate-binding cavity is depicted as a gray surface. Structures correspond to *OvFAD2.4*, *OvFAD2.3*, *OvFAH12*, *OvFAD2.1* and *OvFAD2.5* (*O. violaceus*), *PlFAH12* (*P. lindheimeri*), *DruFAD2* and *DruFAH12* (*D. rufescens*), and *HibFAD2.3*, *HibFAD2.2*, *HibFAD2.1* and *HibFAH12* (*H. benghalensis*). The size of each *FAD* pocket is shown below。

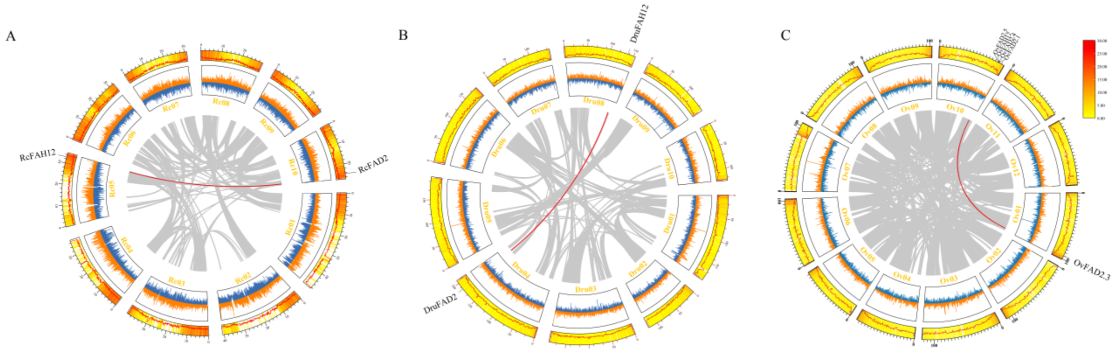

Fig. S9. Synteny analysis of the genomic regions harboring *FAD2* and *FAH12* genes. (A) Circos plot illustrating the microsynteny surrounding the *RcFAD2* and *RcFAH12* loci in the castor bean genome. Circles from outer to inner represent: chromosomal coordinates (scale in megabases), gene density (orange histogram, with peak height proportional to gene number), and GC content (blue heatmap). Gray lines in the background connect systemic blocks within the genome, while the prominent red line highlights the specific segmental duplication event that gave rise to the *FAD2* and *FAH12* gene pair. (B) An analogous synteny analysis of the homologous region in *D. rufescens*, revealing the conserved genomic architecture of the duplicated *FAD2* and *FAH12* genes in a related species. (C) An analogous synteny analysis of the homologous region in *O. violaceus*, revealing the conserved genomic architecture of the duplicated *FAD2* and *FAH12* genes in a related species.

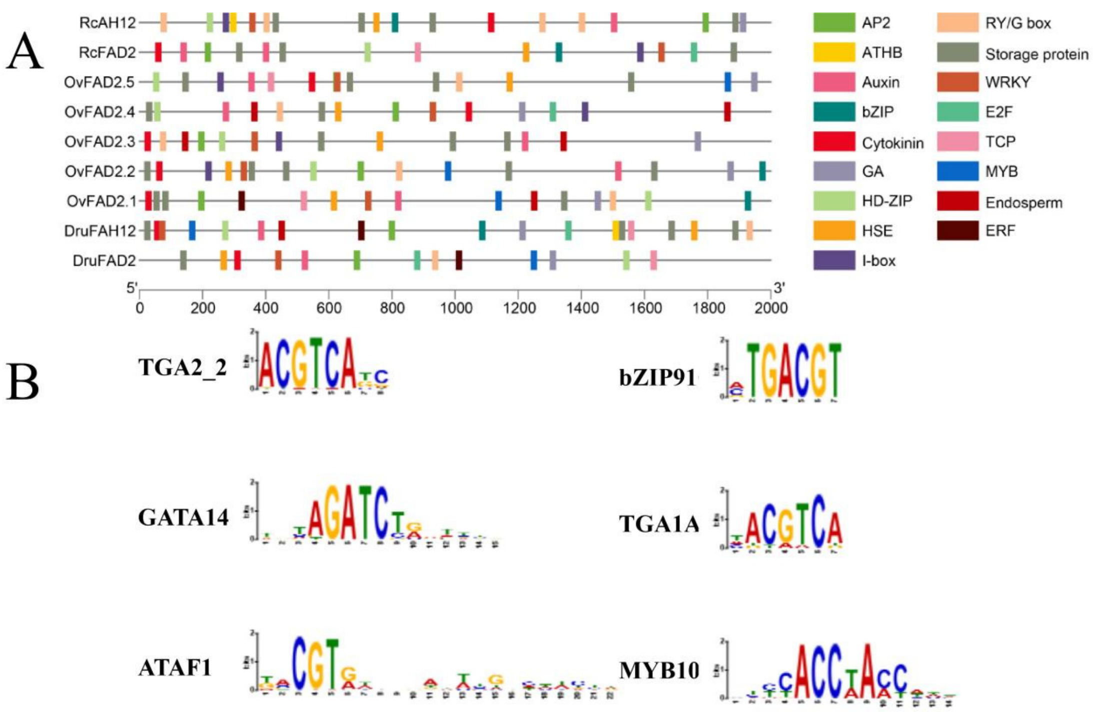

Fig. S10. Analysis of cis-regulatory elements in the promoters of *FAD2* and *FAH12* genes. (A) The diagram depicts the types and positions of predicted cis-regulatory elements within the 2000 bp promoter regions upstream of the transcription start sites (TSS) of *FAD2* and *FAH12* genes from *R. communis* (Rc), *D. rufescens* (Dru), and *O. violaceus* (Ov), different elements are color-coded and involved in various regulatory functions. **(B)** Sequence logos and corresponding position weight matrices (PWMs) of the six significantly enriched conserved motifs identified by SEA analysis.

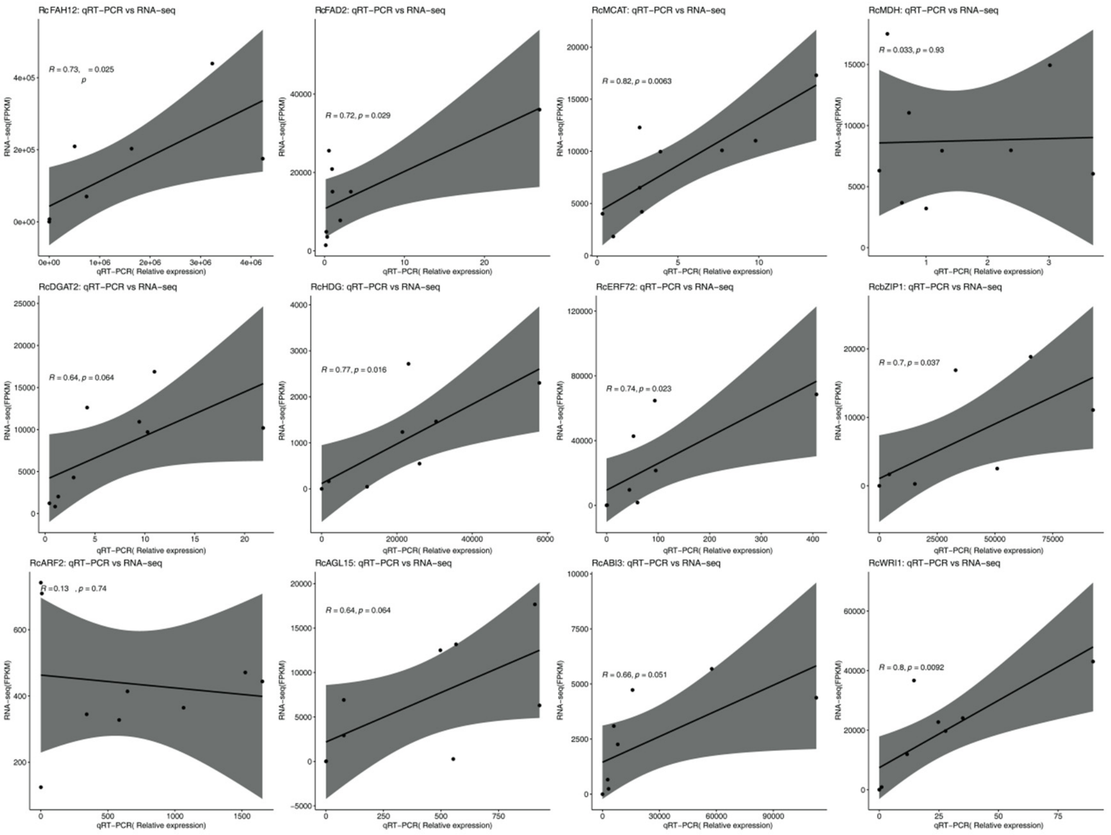

Fig. S11. Correlation between qRT-PCR and RNA-seq expression measurements for selected genes in *R. communis*. Scatter plots showing the correlation between transcript abundances measured by RNA-seq (FPKM) and qRT-PCR (relative expression) across different tissues and seed developmental stages. Each dot represents an independent biological sample. The solid line indicates linear regression, with Pearson correlation coefficients (R) and p-value shown in each panel.
